# Machine learning-assisted network pharmacology reveals that the Chaihu-Longgu-Muli decoction modulates the inflammatory microenvironment to treat perimenopausal syndrome

**DOI:** 10.3389/fmolb.2025.1719463

**Published:** 2025-12-18

**Authors:** Puiian Wong, Ruoyu Li, Ding Li, Bin Fang, Yun Lan, Yuhang Qi, Jiaqian Zheng, Hui Mo

**Affiliations:** 1 Faculty of Chinese Medicine, Macau University of Science and Technology, Taipa, Macau, China; 2 The First Affiliated Hospital of Guangzhou University of Chinese Medicine, Guangzhou, Guangdong, China; 3 The First Clinical Medical College, Guangzhou University of Chinese Medicine, Guangzhou, Guangdong, China

**Keywords:** perimenopausal syndrome, Chaihu-Longgu-Muli decoction, network pharmacology, machine learning, molecular docking, molecular dynamics simulation, inflammation aging

## Abstract

**Background:**

Chaihu-Longgu-Muli decoction (CLMD) is a traditional Chinese medicine formula that shows promise in alleviating symptoms related to premenstrual syndrome (PMS). However, the underlying mechanism remains unclear. This study uses a machine learning-assisted framework integrated with network pharmacology and experimental validation to elucidate the key targets and signaling pathways involved in the therapeutic effects of CLMD on PMS.

**Methods:**

We developed an integrative research framework that incorporates network pharmacology, machine learning, molecular dynamics, and *in vitro* validation. First, we built an overlap network by intersecting disease-related gene sets with data from the TCMSP, BATMAN-TCM, and other relevant databases. We subsequently performed GO and KEGG enrichment analyses. Second, we generated a protein‒protein interaction (PPI) network and screened key targets via machine learning algorithms. Third, we evaluated key active components and targets for ligand‒receptor binding stability via molecular docking and 200 ns MD simulations. Finally, we validated the proposed mechanism by assessing the ability of CLMD to modulate the inflammatory microenvironment using Raw264.7 cells as the experimental model.

**Results:**

By constructing an intersecting network of CLMD-active ingredient-disease targets, we identified 204 representative active components and nearly 300 potential targets. Intersecting these genes with PMS-related genes yielded 46 key targets. The PPI network built in Cytoscape/STRING, together with multiple machine learning algorithms (LASSO, SVM-RFE, and random forest), was used to select key targets, including IL6, TNF, and IL1B. At the molecular level, the key active components (quercetin, kaempferol, and wogonin) showed strong docking affinities to these targets (binding energies <−5.0 kcal/mol) and exhibited stable MD conformations. CLMD intervention significantly downregulated IL6, TNF, and IL1B, reduced reactive oxygen species (ROS) accumulation, and promoted macrophage polarization from the proinflammatory M1 phenotype to the reparative M2 phenotype. Consequently, the experimental findings corroborate the network pharmacology predictions.

**Conclusion:**

CLMD exerts its therapeutic effects through multicomponent-multitarget-multipathway synergy that modulates the inflammatory microenvironment, which provides mechanistic insight into relieving the multidimensional symptoms of PMS.

## Graphical Abstract

**Figure:**
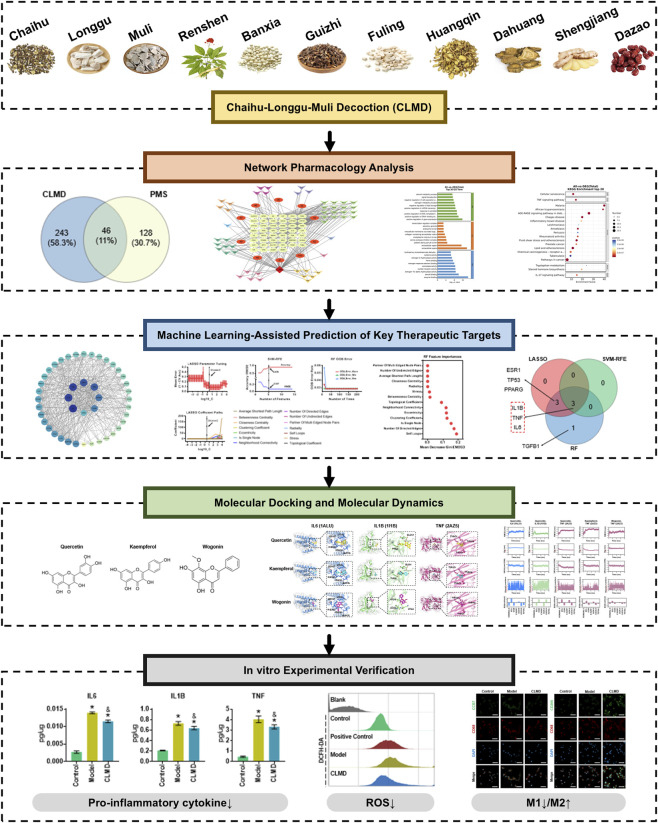

## 1 Introduction

Perimenopausal syndrome (PMS) is a common set of physiological and psychological changes observed in women during the menopausal transition. The condition is marked by fluctuations or decreases in estrogen levels due to progressive ovarian senescence, which in turn provokes a range of autonomic and neuropsychological disturbances. Common manifestations include hot flashes, night sweats, palpitations, insomnia, mood disturbances, and anxiety. These symptoms have the potential to adversely affect quality of life and mental health (Crandall et al., 2023; Uhlig et al., 2019). In addition to its impact on daily functioning, PMS may be associated with longer-term health risks, including cardiovascular disease and osteoporosis, thereby having substantial implications for women’s health and well-being (Inayama et al., 2023; Santoro, 2016). In recent years, the prevalence of PMS has increased, driven by population aging and increasing awareness of women’s health. This phenomenon has led to a growing public health concern (Reeves et al., 2018).

In contemporary medicine, the management of PMS generally involves hormone replacement therapy (HRT), antidepressants, and sedative-hypnotics. Although HRT has been demonstrated to effectively alleviate symptoms associated with menopause, it is associated with certain risks, including an increased incidence of breast cancer, endometrial cancer, and thromboembolic events (Habeshian et al., 2024; Inayama et al., 2023). Moreover, antidepressants and sedative-hypnotics may alleviate certain symptoms, and they can concomitantly induce adverse effects, such as somnolence, dizziness, and dependency (Chen et al., 2023; Wislowska-Stanek et al., 2025). Consequently, the pursuit of safer and more effective treatment strategies has become an important field of current research.

Traditional Chinese medicine (TCM) offers unique advantages in the treatment of menopausal syndrome. It has been widely employed for managing PMS (Wang et al., 2023; Xia et al., 2021). Modern investigations indicate that multiple components of CLMD can modulate endocrinology, alleviate anxiety and depression, and improve sleep, thereby addressing PMS-related symptoms through multiple mechanisms (Li M et al., 2025; Li Z et al., 2025; Wang et al., 2023; Xia et al., 2021). However, the precise mechanisms underlying the therapeutic effects of CLMD on PMS remain incompletely understood.

On the basis of these findings, this study aimed to elucidate the mechanism of CLMD in the treatment of PMS through integrative approaches that combine network pharmacology, machine learning-based prediction, molecular dynamics (MD) simulations, and *in vitro* experiments. The research proceeded in four sequential steps: (1) Network pharmacology analysis to predict the active ingredients of CLMD and identify key targets and signaling pathways involved. (2) Machine learning-assisted prediction and subsequent screening to refine potential therapeutic targets. (3) Validation of the binding stability between CLMD active components and predicted targets via 200 ns MD simulations. (4) *In vitro* experiments to corroborate the computational findings and assess the therapeutic relevance. This research provides a more robust theoretical basis for the clinical application of CLMD and offers new scientific evidence supporting the use of traditional Chinese medicine in the management of menopausal syndrome.

## 2 Methods and materials

### 2.1 Network pharmacology analysis

#### 2.1.1 Collection of the active components of CLMD

To obtain the pharmacologically active ingredients and their targets of Chaihu-Longgu-Muli decoction (CLMD) from two databases, the Traditional Chinese Medicine Systems Pharmacology Database and Analysis Platform (TCMSP) at https://www.tcmsp-e.com/ and BATMAN-TCM at http://bionet.ncpsb.org.cn/batman-tcm/index.php, the following screening criteria were applied: oral bioavailability (OB) ≥ 30, drug-likeness (DL) ≥ 0.18, and half-life (HL) ≥ 4 (Gu et al., 2025). The TCMSP database was then utilized to identify the targets of CLMD. Given that the TCMSP does not contain data regarding Longgu and Muli, in BATMAN-TCM, the search for Longgu and Muli was conducted with the input format set to Herb. The score was set at 20, and the confidence interval was set at *p* < 0.05 to retrieve the active ingredients and targets of Longgu and Muli. Finally, the UniProt database (http://www.uniprot.org/) is employed to harmonize the nomenclature of drug targets, facilitating the identification of pharmacologically active ingredients and their corresponding targets for CLMD.

#### 2.1.2 Screening of disease targets for PMS

To identify disease targets related to perimenopausal syndrome (PMS), searches should be performed via the GeneCards database (http://www.genecards.org/) and DrugBank database (http://www.go.drugbank.com/). The keywords “perimenopausal syndrome” and “PMS” should be used in the searches. The next step is to compile a consolidated list of these disease targets. The aggregated disease targets should subsequently be cross-matched with the pharmacological targets identified earlier for CLMD to obtain potential therapeutic targets for CLMD in treating PMS.

#### 2.1.3 Construction of the network of CLMD-Active ingredients--disease targets

The potential therapeutic targets, along with their corresponding active ingredients and the herbal formula, were entered into Cytoscape 3.9.1 to construct the CLMD-active ingredient–disease target network. In this network, the nodes represent diseases, the formula, active ingredients, and potential targets, whereas the edges represent their interactions. The utilization of Network Analyzer was imperative for the execution of a topological analysis, which is subsequently employed for the identification of key active ingredients and key targets. These ingredients and targets are determined on the basis of metrics such as betweenness centrality, closeness centrality, and degree.

#### 2.1.4 GO and KEGG enrichment analysis

The potential therapeutic targets for treating PMS with CLMD should be imported into the DAVID database (https://david.ncifcrf.gov/home.jsp) to perform functional annotation analyses, including Gene Ontology (GO) enrichment and Kyoto Encyclopedia of Genes and Genomes (KEGG) pathway enrichment.

### 2.2 Machine learning-assisted prediction of key therapeutic targets

#### 2.2.1 Protein‒protein interaction (PPI) network

The potential therapeutic targets for treating perimenopausal syndrome with CLMD were imported into the STRING database (http://string-db.org/). The species was set to *Homo sapiens*, and the protein–protein interaction (PPI) network was retrieved.

#### 2.2.2 Machine learning

The present study utilized the node properties of the PPI network as its foundation. Initially, network features (e.g., closeness centrality, betweenness centrality, etc.) were exported from databases to construct a feature matrix. Subsequent to the elimination of irrelevant columns, the top 20% of genes by degree were designated positives, with the remaining genes assigned as negatives, thereby establishing the binary classification label (Barabási and Albert, 1999; Jeong et al., 2001; Yu et al., 2007). Consequently, with the exception of degree, all network features are standardized. The dataset is partitioned into a training set and a testing set at a ratio of 8:2 (Xiaoying et al., 2025). The training set is utilized for model training, whereas the testing set is employed to score and rank all the genes.

Three machine learning algorithms from the scikit-learn library are employed to calculate scores for each gene: L1-regularized logistic regression (LASSO), support vector machine-recursive feature elimination (SVM-RFE), and random forest (RF) (Qiao et al., 2025). The average of these scores is subsequently calculated to yield a composite score. The ranking of genes is determined by this composite score, with the highest-scoring candidates being selected.

### 2.3 Molecular docking and molecular dynamics

#### 2.3.1 Molecular docking verification

In accordance with the predictions of fundamental targets derived from machine learning algorithms, the crystal structures of said targets are retrieved from the Protein Data Bank (PDB) database (https://www.rcsb.org/). To validate interactions and predict the binding interactions between the key proteins and the key ingredient ligands, it is necessary to employ AutoDock Vina (http://vina.scripps.edu/AutoDock) to dock the target proteins with key small-molecule compounds.

#### 2.3.2 Molecular dynamics simulation

The structural complexes were prepared via GROMACS 2024-2 (Pronk et al., 2013). The GROMACS engine serves as the backend, with a common simulation setting applied to all complexes. This configuration enables the execution of high-throughput MD simulations. The pipeline under consideration comprises four stages: preparation, minimization, equilibration, and production simulations. In the system preparation stage, a protein topology is generated with pdb2gmx, using the amber14sb and the tip3p explicit water model (Li S et al., 2025; Zhang et al., 2025). A cubic simulation box is subsequently employed, with a minimum distance of 1.2 nm maintained between the complex and the box boundaries. Subsequent to the preparation of the simulation box, an energy minimization process is initiated to eliminate atomic clashes and optimize the geometry of all the molecules. In the equilibration simulation stage, a thermostat is employed to increase the system’s temperature from 0 to 300 K within a span of 100 ps. The system is then equilibrated to 1 bar in an NPT ensemble for an additional 100 ps. During the equilibration stage, the bonds for molecules are constrained. In the final production iteration, the leap-frog algorithm is employed to integrate Newton’s equations of motion, whereas the particle mesh Ewald (PME) method is utilized to calculate long-range electrostatic interactions. The limited-memory Broyden-Fletcher-Goldfarb-Shanno (LINCS) algorithm is adopted for resetting all bonds to their correct lengths after an unconstrained update. Subsequent to the relaxation phase, the systems were subjected to 200 ns production simulations with an integration time step of 2 fs. Finally, the data included the root mean square deviation (RMSD), radius of gyration (Rg), solvent accessible surface area (SASA), hydrogen bond number, and change in free energy (ΔG) (Elekofehinti et al., 2021).

### 2.4 *In vitro* experimental verification

#### 2.4.1 Preparation of CLMD

The experimental preparation, designated CLMD, consists of the following ingredients: Bupleurum (Chaihu) 12 g, Fossilia ossis mastodi (Longgu) 4.5 g, Oyster gigas (Muli) 4.5 g, Panax ginseng (Renshen) 4.5 g, Scutellaria baicalensis (Huangqin) 4.5 g, Pinellia ternata (Banxia) 4.5 g, Cinnamon Twig (Guizhi) 4.5 g, Poria (Fuling) 4.5 g, Rheum palmatum (Dahuang) 6 g, Zingiber officinale (Shengjiang) 4.5 g, and Ziziphus jujuba (Dazao) 6 g (approximately 18 g). The aforementioned Chinese medicinal materials are immersed in 400 mL of water for extraction and subsequently simmered until the volume is reduced to 100 mL. The solution was subsequently filtered through a 0.22 μm filter and then subjected to lyophilization. In this study, the intervention concentrations of CLMD were calculated on the basis of the crude drug mass and applied at the following concentrations: low concentration (3.6 μg/mL), medium concentration (36 μg/mL), and high concentration (360 μg/mL).

#### 2.4.2 Cell culture

The RAW264.7 cell line present in this research was obtained from Pricella Biotechnology Co., Ltd. (China). And was cultivated in DMEM containing 10% fetal bovine serum and 1% penicillin‒streptomycin at 37 °C in a 5% CO_2_ atmosphere.

#### 2.4.3 CCK-8

RAW264.7 cells were seeded in 6-well plates. After 24 h, the LPS and CLMD were added to the designated wells. At 24 h, 48 h, and 96 h postintervention, cell viability was evaluated via a CCK-8 kit. The optical density (OD) value was subsequently measured at 450 nm.

#### 2.4.4 ELISA

RAW264.7 cells were seeded in 6-well plates. Following the 24 h incubation period, the cells were treated with LPS and CLMD. Following an additional 48 h of incubation, the cell samples were collected. The concentrations of IL6, IL1B, and TNF were measured via an ELISA kit (Cloud-Clone, China) following the manufacturer’s instructions. The total protein content was determined via a Bradford protein assay kit (Solarbio, China).

#### 2.4.5 Reactive oxygen species (ROS)

RAW264.7 cells were seeded in 6-well plates. Following a 24 h period of incubation, the cells were treated with LPS and CLMD. Following an additional 48 h of cultivation, reactive oxygen species (ROS) levels were evaluated in accordance with the protocol outlined in the ROS detection kit (Beyotime, China). In accordance with the instructions provided by the kit, the samples were incubated in the dark for 1 hour prior to detection by flow cytometry (FCM).

#### 2.4.6 Immunofluorescence (IF)

RAW264.7 cells were inoculated into confocal culture dishes. After 24 h of cultivation, LPS and CLMD were added. The cultivation was continued for another 48 h. Then, IF staining was carried out according to conventional procedures. Finally, the cells were observed via confocal microscopy. The following antibodies were used: CCR7 (AF5293, Affinity, United States), CD206 (DF4149, Affinity, United States), and CD68 (AB53444, Abcam, United States).

#### 2.4.7 RT‒qPCR

RAW264.7 cells were inoculated into confocal culture dishes. After 24 h of cultivation, LPS and CLMD were added. The cells were further cultivated for another 48 h. Then, the cell samples were collected, and total RNA was extracted for PCR experiments. GAPDH was used as the internal reference. The relative mRNA expression level of the target gene was calculated via the 2^−ΔΔCt^ method. Primer sequences used in this research were listed in Table 1.

**TABLE 1:** Primer sequences.

Genes	Sequences (5′-3′)
TNF	F: ATGTCTCAGCCTCTTCTCATTC R: GCTTGTCACTCGAATTTTGAGA
IL1B	F: CACTACAGGCTCCGAGATGAACAAC R: TGTCGTTGCTTGGTTCTCCTTGTAC
IL6	F: CTCCCAACAGACCTGTCTATAC R: CCATTGCACAACTCTTTTCTCA
IL4	F: CCATATCCACGGATGCGACA R: AAGCCCGAAAGAGTCTCTGC
IL10	F: CAGTACAGCCGGGAAGACAA R: GACACCTTGGTCTTGGAGCTTA
TGFβ	F: GACCTGGGTTGGAAGTGGAT R: TTGGTTGTAGAGGGCAAGGA
GAPDH	F: GTTCCAGTATGACTCCACTC R: CCTCACCCCATTTGATGTTA

#### 2.4.8 Statistical analysis

The data are expressed as the mean ± standard deviation (SD). All quantitative data were obtained with at least three replicates. Statistical analyses were performed with GraphPad Prism 10.0. Data were analyzed by one-way analysis of variance (ANOVA) followed by Fisher’s LSD test for *post hoc* pairwise comparisons. A significance level of *p* < 0.05 is indicative of a statistically significant difference between groups.

## 3 Results

### 3.1 Potential mechanism of CLMD in treating PMS

**FIGURE 1:**
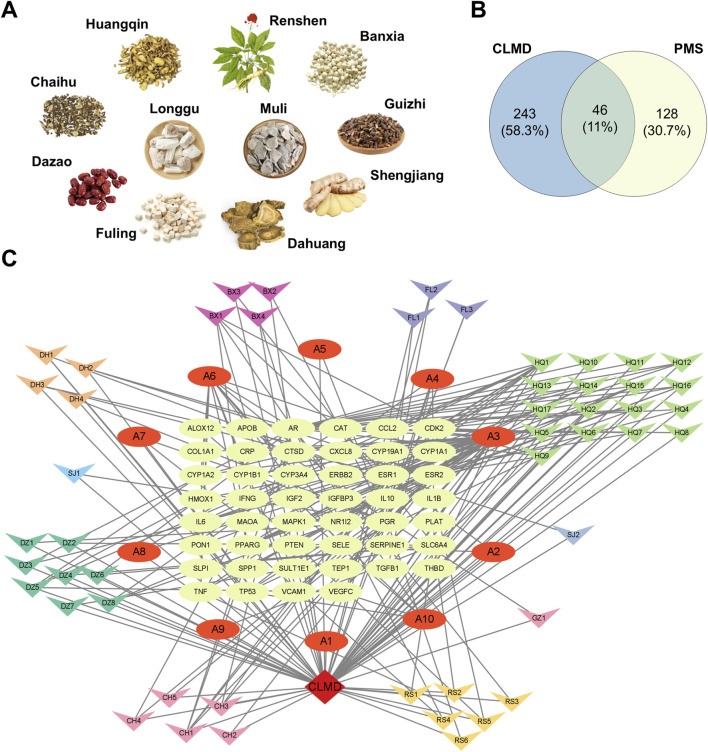
Therapeutic Targets of CLMD for PMS Treatment. **(A)** The herbal components of CLMD. **(B)** Venn diagram of the intersections of the CLMD targets and PMS-related targets. **(C)** Network diagram of CLMD-active ingredients-disease targets. Notes: CLMD, Chaihu-Longgu Muli decoction.

The herbal components of Chaihu-Longgu-Muli decoction (CLMD) are shown in Figure 1A. Using the TCMSP database, we screened active ingredients with the following criteria: oral bioavailability (OB) ≥ 30%, drug-likeness (DL) ≥ 0.18, and a half-life (HL) ≥ 4. This yielded 160 active ingredients (Table 2). Further analysis eliminated 41 ingredients lacking relevant targets, resulting in 119 active ingredients linked to 266 targets. Additionally, 5 active ingredients and 27 related targets were retrieved for Longgu and Muli from the BATMAN-TCM database (Table 2). Concurrently, 174 PMS-related targets were obtained from the GeneCards and DrugBank databases. By intersecting the CLMD targets with the PMS disease targets, 46 effective targets were identified (Figure 1B).

**TABLE 2:** Information on the effective active ingredients of CLMD.

No.	Identifier	Ingredient	OB (%)	DL	Source
MOL000073	A1	Ent-epicatechin	48.96	0.24	Guizhi, Huangqin
MOL000096	A2	(−)-Catechin	49.68	0.24	Dahuang, Dazao
MOL000098	A3	Quercetin	46.43	0.28	Chaihu, Dazao
MOL000358	A4	Beta-sitosterol	36.91	0.75	Banxia, Dahuang, Dazao, Guizhi, Huangqin, Renshen, Shengjiang
MOL000359	A5	Sitosterol	36.91	0.75	Guizhi, Huangqin
MOL000422	A6	Kaempferol	41.88	0.24	Chaihu, Renshen
MOL000449	A7	Stigmasterol	43.83	0.76	Banxia, Chaihu, Dazao, Huangqin, Renshen, Shengjiang
MOL000492	A8	(+)-Catechin	54.83	0.24	Dazao, Guizhi
MOL000787	A9	Fumarine	59.26	0.83	Dazao, Renshen
MOL002714	A10	Baicalein	33.52	0.21	Banxia, Huangqin
MOL000519	BX1	Coniferin	31.11	0.32	Banxia
MOL001755	BX2	24-Ethylcholest-4-en-3-one	36.08	0.76	Banxia
MOL002670	BX3	Cavidine	35.64	0.81	Banxia
MOL006957	BX4	(3S,6S)-3-(benzyl)-6-(4-hydroxybenzyl)piperazine-2,5-quinone	46.89	0.27	Banxia
MOL000354	CH1	Isorhamnetin	49.6	0.31	Chaihu
MOL000490	CH2	Petunidin	30.05	0.31	Chaihu
MOL004598	CH3	3,5,6,7-Tetramethoxy-2-(3,4,5-trimethoxyphenyl)chromone	31.97	0.59	Chaihu
MOL004609	CH4	Areapillin	48.96	0.41	Chaihu
MOL004718	CH5	A-spinasterol	42.98	0.76	Chaihu
MOL000471	DH1	Aloe-emodin	83.38	0.24	Dahuang
MOL002235	DH2	EUPATIN	50.8	0.41	Dahuang
MOL002281	DH3	Toralactone	46.46	0.24	Dahuang
MOL002297	DH4	Daucosterol_qt	35.89	0.7	Dahuang
MOL000211	DZ1	Mairin	55.38	0.78	Dazao
MOL000627	DZ2	Stepholidine	33.11	0.54	Dazao
MOL001454	DZ3	Berberine	36.86	0.78	Dazao
MOL001522	DZ4	(S)-Coclaurine	42.35	0.24	Dazao
MOL002773	DZ5	Beta-carotene	37.18	0.58	Dazao
MOL007213	DZ6	Nuciferin	34.43	0.4	Dazao
MOL012921	DZ7	Stepharine	31.55	0.33	Dazao
MOL012976	DZ8	Coumestrol	32.49	0.34	Dazao
MOL000282	FL1	Ergosta-7,22e-dien-3beta-ol	43.51	0.72	Fuling
MOL000283	FL2	Ergosterol peroxide	40.36	0.81	Fuling
MOL000296	FL3	Hederagenin	36.91	0.75	Fuling
MOL004576	GZ1	Taxifolin	57.84	0.27	Guizhi
MOL000173	HQ1	Wogonin	30.68	0.23	Huangqin
MOL000228	HQ2	(2R)-7-hydroxy-5-methoxy-2-phenylchroman-4-one	55.23	0.2	Huangqin
MOL000525	HQ3	Norwogonin	39.4	0.21	Huangqin
MOL000552	HQ4	5,2′-Dihydroxy-6,7,8-trimethoxyflavone	31.71	0.35	Huangqin
MOL001458	HQ5	Coptisine	30.67	0.86	Huangqin
MOL001689	HQ6	Acacetin	34.97	0.24	Huangqin
MOL002897	HQ7	Epiberberine	43.09	0.78	Huangqin
MOL002909	HQ8	5,7,2,5-Tetrahydroxy-8,6-dimethoxyflavone	33.82	0.45	Huangqin
MOL002917	HQ9	5,2′,6′-Trihydroxy-7,8-dimethoxyflavone	45.05	0.33	Huangqin
MOL002925	HQ10	5,7,2′,6′-Tetrahydroxyflavone	37.01	0.24	Huangqin
MOL002927	HQ11	Skullcapflavone II	69.51	0.44	Huangqin
MOL002928	HQ12	Oroxylin a	41.37	0.23	Huangqin
MOL002932	HQ13	Panicolin	76.26	0.29	Huangqin
MOL002933	HQ14	5,7,4′-Trihydroxy-8-methoxyflavone	36.56	0.27	Huangqin
MOL002934	HQ15	Neobaicalein	104.34	0.44	Huangqin
MOL008206	HQ16	Moslosooflavone	44.09	0.25	Huangqin
MOL012266	HQ17	Rivularin	37.94	0.37	Huangqin
MOL003648	RS1	Inermin	65.83	0.54	Renshen
MOL005308	RS2	Aposiopolamine	66.65	0.22	Renshen
MOL005317	RS3	Deoxyharringtonine	39.27	0.81	Renshen
MOL005321	RS4	Frutinone A	65.9	0.34	Renshen
MOL005344	RS5	Ginsenoside rh2	36.32	0.56	Renshen
MOL005399	RS6	Alexandrin_qt	36.91	0.75	Renshen
MOL001771	SJ1	Poriferast-5-en-3beta-ol	36.91	0.75	Shengjiang
MOL006129	SJ2	6-methylgingediacetate2	48.73	0.32	Shengjiang
\	\	Aluminum	\	\	Muli
\	\	Calcium sulphate	\	\	Muli
\	\	Calcium phosphate	\	\	Longgu, Muli
\	\	Calcium carbonate	\	\	Longgu, Muli
\	\	Silicon	\	\	Muli

The intersecting network of CLMD-active ingredients-disease targets is illustrated in Figure 1C. This network had 107 nodes and 232 edges. The green nodes represent targets. Red elliptical nodes denote active ingredients common to two or more herbs. I Various colored inverted triangle nodes indicate effective active ingredients. After performing the network analysis, we listed the top 10 active ingredients and targets by degree centrality (Table 3).

**TABLE 3:** The intersecting network of CLMD-active ingredients-disease targets of key active ingredients and targets.

Ingredient	Degree	Target	Degree
Quercetin	35	Androgen receptor, AR	29
Kaempferol	14	Estrogen receptor 1, ESR1	14
Wogonin	11	Progesterone receptor, PGR	13
Isorhamnetin	6	Peroxisome proliferator activated receptor gamma, PPARG	12
Baicalein	6	Estrogen receptor 2, ESR2	12
Beta-sitosterol	5	Solute carrier family 6 member 4, SLC6A4	10
Coniferin	5	Cyclin dependent kinase 2, CDK2	9
Acacetin	5	Tumor protein P53, TP53	5
Beta-carotene	5	Cytochrome P450 family 1 subfamily a member 2, CYP1A2	4
5,7,4′-Trihydroxy-8-methoxyflavone	5	Cytochrome P450 family 3 subfamily a member 4, CYP3A4	4

**FIGURE 2:**
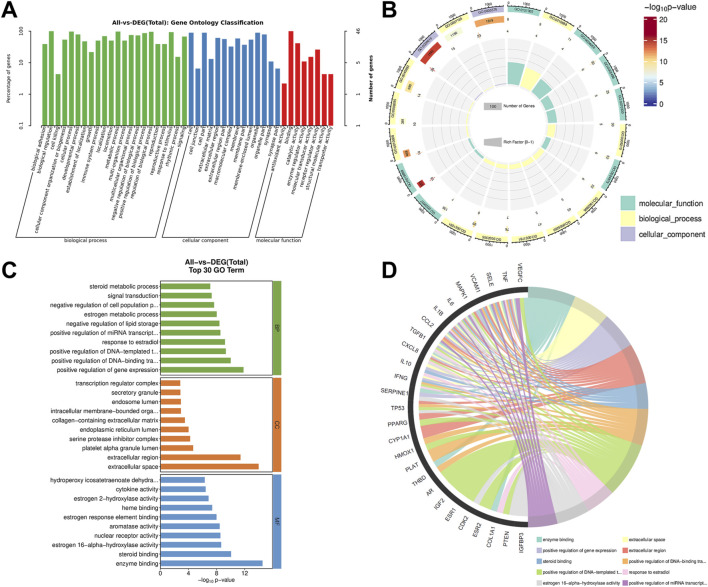
GO enrichment analysis. **(A)** GO enrichment across the three ontologies: BP, CC, and MF. **(B)** Circos plot illustrating the GO enrichment results. **(C)** The top 10 enriched GO terms for BP, CC, and MF. **(D)** Chord diagram of the GO term enrichment network. Notes: GO enrichment: Gene Ontology enrichment, BP, biological process, CC, cellular component, MF, molecular function.

GO enrichment analysis was performed on the targets of CLMD treatment for PMS. The enrichment results revealed that there were 1,285 GO enrichment terms, including 973 biological process (BP) terms, 119 cellular component (CC) terms, and 193 molecular function (MF) terms. Among the BP terms, biological regulation, cellular process, and developmental process had the most enriched genes, with 46, 45, and 36 genes, respectively (Figure 2A). Among the CC categories, the terms “membrane” and “organelle” were the most enriched (Figure 2A). In the MF category, binding and catalytic activity were the most enriched (Figure 2A). Further analysis via a Circos plot revealed that estrogen 16-alpha-hydroxylase activity (GO:0101020), estrogen response element binding (GO:0034056), aromatase activity (GO:0070330), and estrogen metabolic process (GO:0008210) were significantly enriched (Figure 2B). The top 10 BP, CC, and MF terms were selected on the basis of the count value and are displayed in Figure 2C. Notably, in the BP category, positive regulation of gene expression and positive regulation of DNA-binding transcription factor activity and response to estradiol were significant, suggesting that gene expression and estrogen-related regulation are core processes. Finally, a chord diagram was used to display the relationships between the enriched genes and the enriched GO terms. These results suggested that TGFB1, TNF, AR, ESR1, PPARG, IL10, IL6, and IL1B are involved in the important BP, CC, and MF pathways of CLMD regulation (Figure 2D).

**FIGURE 3:**
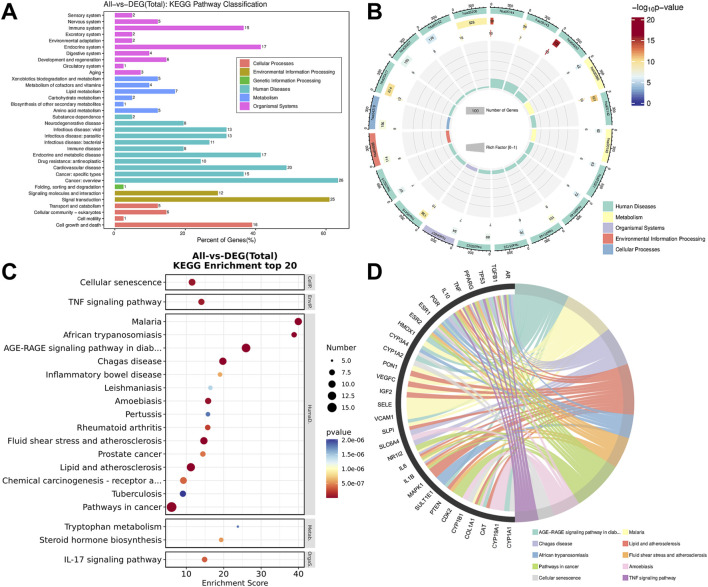
KEGG enrichment analysis. **(A)** KEGG enrichment at classification. **(B)** Circos plot analyzing the KEGG enrichment results. **(C)** The top 20 enriched KEGG pathways. **(D)** Chord diagram of the KEGG pathway enrichment network. Notes: KEGG enrichment: Kyoto Encyclopedia of Genes and Genomes enrichment.

We performed KEGG enrichment analyses on the CLMD targets for treating PMS and classified the enriched terms according to the following categories: Human Diseases (HumanD.), metabolism (Metab.), organismal systems (OrgaS.), Environmental Information Processing (EnIP), and Cellular Processes (CellP) (Figure 3A). The enrichment results revealed that terms such as “cancer overview” (HumanD.), signal transduction (EnIP), cardiovascular disease (HumanD.), endocrine and metabolic disease (HumanD.), and endocrine system (OrgaS.) The most enriched genes included 26, 25, 20, 17, and 17 genes, respectively (Figure 3A). Furthermore, the Circos results depicted the enrichment more vividly. Categories such as malaria (hsa05144) and African trypanosomiasis (hsa05143) contained a greater number of enriched genes and presented significant *p* values (Figure 3B). We selected the top 20 enriched KEGG terms, including “cellular senescence” and “TNF signaling pathway” (Figure 3C). Finally, the chord diagram illustrates the correspondence between enriched genes and KEGG terms (Figure 3D). The results suggested that differentially expressed genes (DEGs), including IL6, TNF, IL1B, CXCL8, TGFB1, SELE, VCAM1, MAPK1, and CCL2, participated in key CLMD-regulated KEGG pathways (Figure 3D).

### 3.2 Machine learning-assisted identification and screening of key targets

**FIGURE 4:**
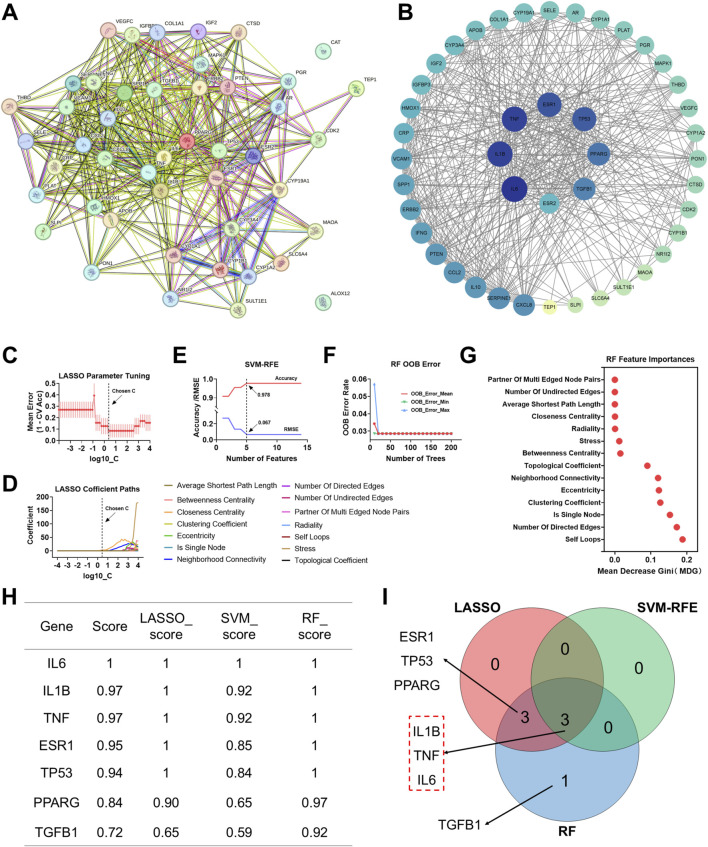
Machine Learning-Assisted Identification and Screening of Key Targets. **(A)** PPI network diagram. **(B)** High-degree genes of the PPI network. **(C)** Parameter tuning and coefficient path curve of LASSO. **(D)** Coefficient path curve of LASSO. **(E)** Accuracy and error curves of SVM-RFE. **(F)** OOB error curve of the RF. **(G)** The feature importance of the RF based on the MDG. **(H)** The prediction results of LASSO, SVM-RFE and RF. **(I)** Venn diagram showing the overlap of the prediction targets of LASSO, SVM-RFE and RF. Notes: PPI network: protein‒protein interaction network, LASSO, L1-regularized logistic regression, SVM-RFE, support vector machine-recursive feature elimination, RF, random forest.

We uploaded the shared targets to the STRING platform for analysis and constructed the PPI network. Each node represents a target. The colors and sizes of the nodes ranged from dark to light and from large to small, respectively, to reflect decreasing target degrees. Edges indicate interactions between two targets (Figures 4A,B). Using Network Analyzer, we found that the PPI network had an average degree of 40.36 and an average betweenness centrality of 1.29 × 10^−2^. Eight targets exceeded these averages in both degree centrality and betweenness centrality. These findings suggested that IL6, IL1B, TNF, ESR1, TP53, and three other targets may be key to treating PMS (Figure 4B; Table 4).

**TABLE 4:** The key targets in the PPI network.

No.	Target	Degree	Betweenness centrality	Closeness centrality
1	IL6	82	0.080 318 53	0.955 555 55
2	IL1B	78	0.061 865 10	0.914 893 61
3	TNF	78	0.061 865 10	0.914 893 61
4	ESR1	72	0.061 152 51	0.86
5	TP53	70	0.073 671 30	0.843 137 25
6	PPARG	62	0.023 229 49	0.781 818 18
7	TGFB1	58	0.015 536 16	0.754 385 96
8	ERS2	42	0.014 661 55	0.661 538 46

To further validate the accuracy of the PPI-screened targets, we applied three machine learning algorithms (LASSO, SVM-RFE, and RF) to the top 20° genes as sample data to screen the targets. First, we show the parameter tuning curve and coefficient path curve for the LASSO algorithms (Figure 4C). When the inverse regularization strength (C) was 0.4, the average error rate reached a minimum and remained stable within the 0.4–2.5 interval, confirming 0.4 as the suitable C value for subsequent computations (Figure 4C). According to the LASSO coefficient paths, at C = 0.4, only stress and closeness centrality presented corresponding coefficients of 2.624 and 2.307, respectively (Figure 4D). Therefore, we selected these two features for LASSO-based machine learning prediction. The SVM-RFE accuracy and error curves are shown in Figure 4E. The results indicated that as the number of features increased to five, the SVM-RFE model accuracy reached a maximum of 0.978, whereas the error rate decreased to a minimum of 0.067. We present the RF out-of-bag (OOB) error curve and feature importance (Figure 4F). When the number of features reached 20, the OOB error curve fell to its minimum and stabilized. The feature importance bar chart displays the mean decrease in the Gini (MDG) coefficient of 14 types in the RF algorithm (Figure 4G). The partner of multiedged node pairs was the most important among them (Figure 4G).

The machine learning results across the three algorithms indicated that LASSO predicted 6 potential key targets (ESR1, IL-1B, TNF, TP53, PPARG, and IL-6) with scores greater than 0.9 (Figures 4H,I). SVM-RFE identified IL6, TNF, and IL1B with scores greater than 0.9 (Figures 4H,I). RF identified 7 potential key targets (ESR1, IL1B, TNF, TP53, IL6, PPARG, and TGFB1) with scores greater than 0.9 (Figures 4H,I). The Venn diagram revealed that the intersection of targets across the algorithms included IL6, IL1B and TNF.

### 3.3 MD simulation of the key active ingredients with the key target proteins

**FIGURE 5:**
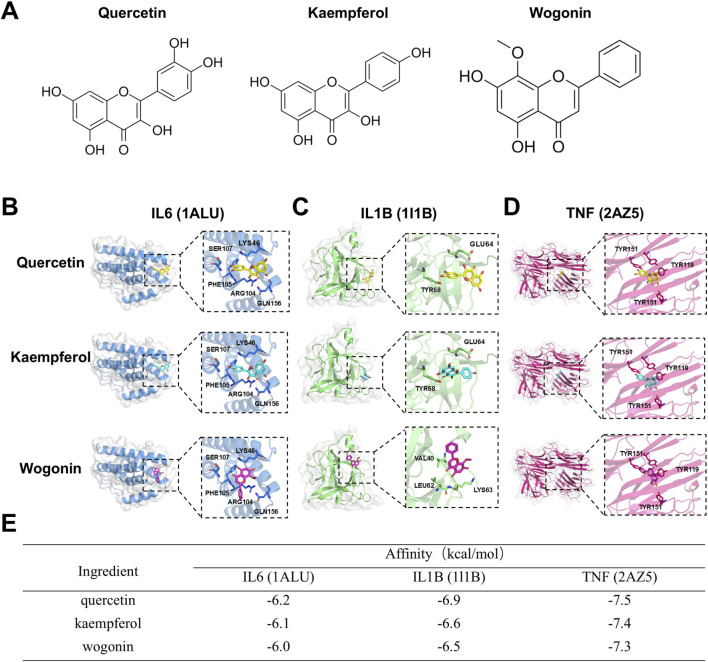
Molecular docking between key active ingredients in CLMD and the key targets selected by machine learning. **(A)** Molecular formulas of quercetin, kaempferol, and wogonin. **(B)** Quercetin docking with IL6, IL1B, and TNF. **(C)** Kaempferol docking with IL6, IL1B, and TNF. **(D)** Wogonin docking with IL6, IL1B, and TNF. **(E)** Binding energy table showing interactions between key active ingredients in CLMD and the key targets selected by machine learning.

We performed molecular docking to validate the interactions between the key active ingredients in CLMD (quercetin, kaempferol, and wogonin) and the key targets selected by machine learning (IL6, IL1B, and TNF). First, we identified the molecular formulas of quercetin, kaempferol, and wogonin (Figure 5A). The docking results revealed that the binding energies between these key active ingredients and the key targets were all less than −5.0 kcal/mol, indicating favorable binding. Specifically, the binding energies of quercetin, kaempferol, and wogonin with IL6 were −6.2, −6.1, and −6.0 kcal/mol, respectively (Figures 5B,E). With respect to IL1B, they were −6.9, −6.6, and −6.5 kcal/mol, respectively (Figures 5C,E). With respect to TNF, they were −7.5, −7.4, and −7.3 kcal/mol, respectively (Figures 5D,E). These binding energies were all less than −5.0 kcal/mol, indicating significant affinity between these ligands and their receptor proteins (Gong et al., 2025).

To further evaluate the stability of the complexes formed after molecular docking, we performed 200 ns MD simulations on representative complexes, including Quercetin-IL6, Quercetin-IL1B, Quercetin-TNF, Kaempferol-TNF, and Wogonin-TNF. We analyzed the root mean square deviation (RMSD), radius of gyration (Rg), solvent accessible surface area (SASA), number of hydrogen bonds numbers, and change in free energy (ΔG). RMSD analysis revealed that the aforementioned complexes achieved a stable state with no significant fluctuations within 50 ns, and their RMSD values remained low. For example, the size of the quercetin-IL6 complex fluctuated between approximately 0.15 and 0.35 nm, approaching stability after 25 ns and suggesting stable binding throughout the simulation. Rg analyses revealed that the overall protein structure did not undergo significant changes during the simulations. The quercetin-IL6 complex had an Rg of approximately 1.4 nm, and the quercetin-IL1B complex stabilized at approximately 1.5 nm. The Rg values of the complexes of quercetin and kaempferol with TNF gradually decreased and stabilized near 2.4 nm. In contrast, the wogonin-TNF complex stabilized at approximately 2.8 nm. SASA analyses revealed that ligand binding did not result in significant changes in the SASA of the target proteins. This suggested that ligand binding had a minimal effect on the overall protein structure. H-bond analysis revealed that the number of hydrogen bonds gradually stabilized in the latter stages of the simulations, with the average value remaining above 2.

**FIGURE 6:**
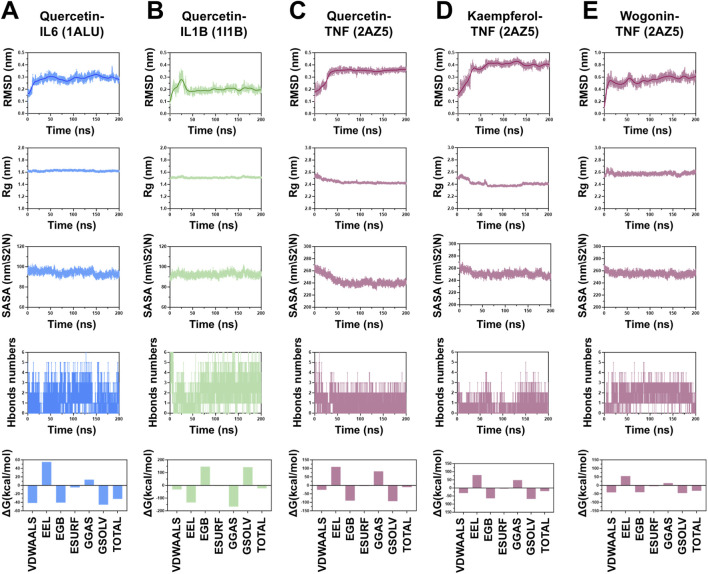
Molecular Dynamics Analysis of the Binding Between Key Active Ingredients in CLMD and the Key Targets Selected by Machine Learning in RMSD, Rg, SASA, and ΔG. **(A)** Quercetin–IL6 complex. **(B)** Quercetin–IL1B complex. **(C)** Quercetin–TNF complex. **(D)** Kaempferol–TNF complex. **(E)** Wogonin–TNF complex. Notes: RMSD, root mean square deviation; Rg, radius of gyration; SASA, solvent accessible surface area; ΔG, change in free energy.

Finally, we applied the MM/GBSA method to calculate the binding free energies, revealing contributions from van der Waals (VDW), electrostatic (EEL), polar solvation (EGB), nonpolar solvation (ESURF), gas-phase energy (GGAS), and solvation energy (GSOLV) interactions. Residue-level contributions with absolute values greater than 1.0 kcal/mol were noted as having significant impacts on complex stability. The total binding energies for the complexes were as follows: quercetin-IL6 (−31.39 ± 2.19 kcal/mol), quercetin-IL1B (−23.23 ± 3.07 kcal/mol), quercetin-TNF (−10.09 ± 2.94 kcal/mol), kaempferol-TNF (−19.54 ± 2.96 kcal/mol), and wogonin-TNF (−31.93 ± 2.19 kcal/mol) (Figure 6).

### 3.4 Verification of the regulatory effect of CLMD on key target proteins

**FIGURE 7:**
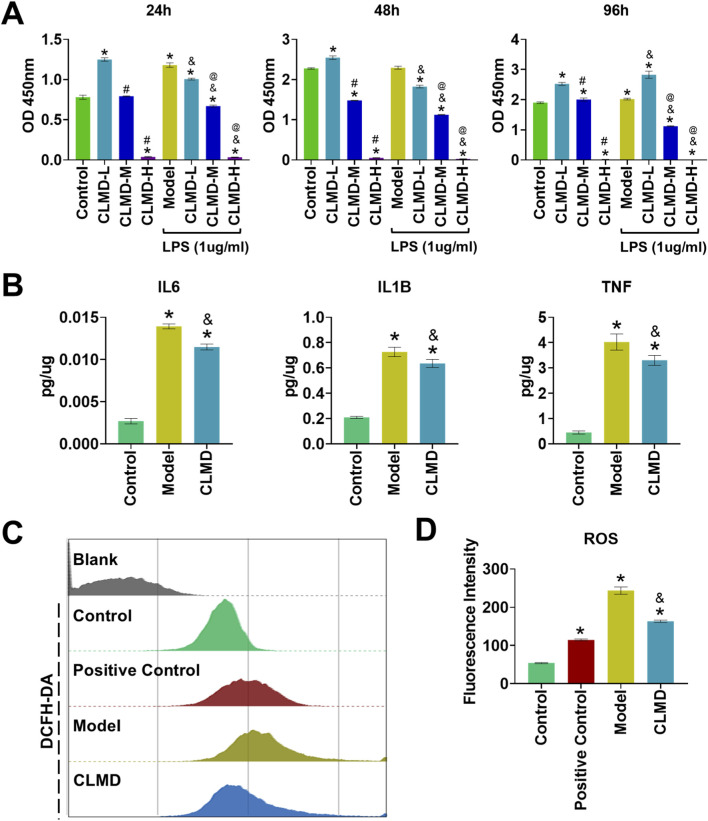
Anti-inflammatory and Antioxidant Effects of CLMD. **(A)** CCK-8 results at 24, 48, and 96 h **(B)** ELISA tests for IL6, IL1B, and TNF. **(C)** FCM analysis of ROS levels. **(D)** Statistical analysis of the fluorescence intensity of ROS. Notes: *p < 0.05 compared with the control group. ^#^ *p* < 0.05 compared with the CLMD-L group. ^&^ *p* < 0.05 compared with the model group. ^@^ *p* < 0.05 compared with the CLMD-L group after LPS intervention.

This research used Raw 264.7 cells to validate the regulatory effects of CLMD on key targets and its potential mechanism. First, a cytotoxicity assay was performed to confirm the appropriate concentration for cell intervention. The CCK-8 results revealed that CLMD significantly promoted Raw 264.7 cell proliferation without causing cytotoxicity when it was administered at a low concentration (3.6 μg/mL) for 24, 48, or 96 h under normal conditions (Figure 7A). However, medium and high concentrations of CLMD exhibited strong cytotoxicity. Therefore, subsequent experiments used a low concentration (3.6 μg/mL) as the intervention condition, which would better distinguish the toxic effects and pharmacological effects of CLMD to reveal its positive biological regulatory mechanisms (Figure 7A).

Additionally, we evaluated the cytotoxicity of cell proliferation under LPS stimulation. LPS is commonly used to induce inflammatory responses in Raw264.7 cells (Deng et al., 2025). The CCK-8 results revealed that, compared with the normal control, the model group (LPS-treated) exhibited increased Raw 264.7 cell proliferation. This inflammation-associated abnormal proliferation is accompanied by the release of numerous inflammatory factors, such as IL6, IL1B and TNF (Deng et al., 2025; Tang et al., 2023). Notably, low-concentration CLMD intervention significantly alleviated the abnormal proliferation induced by LPS in the short term (Figure 7A).

The ELISA results revealed that, compared with those in the control group, the protein expression of IL6, IL1B and TNF-α in the model group (LPS-treated) was significantly greater (*p* < 0.05). These results indicate that the LPS intervention regimen effectively induced an inflammatory response in Raw264.7 cells. CLMD intervention significantly alleviated the increases in the protein levels of IL6, IL1B and TNF induced by LPS compared with those in the model group (Figure 7B). This result was statistically significant (*p* < 0.05). Additionally, the FCM results revealed that the fluorescence intensity decreased significantly after CLMD intervention compared with that in the model group. These results demonstrated that CLMD effectively reduces LPS-induced intracellular ROS accumulation and enhances the antioxidant stress capacity (Figure 7C).

**FIGURE 8:**
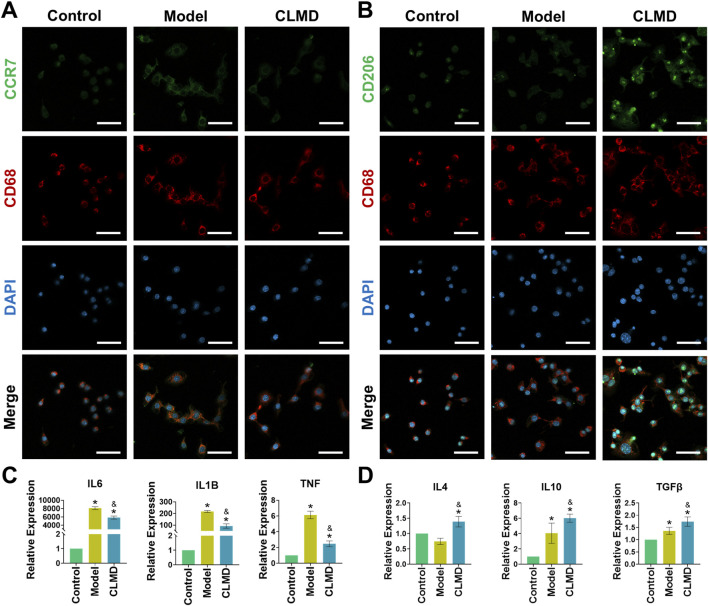
CLMD modulates the polarization phenotype of Raw264.7 cells. **(A)** IF staining of CCR7. **(B)** IF staining of CD206. **(C)** Relative mRNA expression of IL6, IL1B, and TNF. **(D)** Relative mRNA expression of IL4, IL10, and TGFβ. Notes: *p < 0.05 compared with the control group and ^&^ *p* < 0.05 compared with the model group.

CCR7 and CD206 are markers used to identify M1 and M2 phenotype polarization, respectively (Liao et al., 2025; Zhao et al., 2018). Compared with those in the control group, the fluorescence intensity of CCR7 in the model group (LPS intervention) was significantly greater (Figure 8A). These findings indicate that LPS intervention effectively promotes M1 phenotype polarization in Raw264.7 cells. In contrast, CLMD intervention markedly reduced the CCR7 fluorescence intensity compared with that in the model group, suggesting that CLMD inhibits M1 phenotype polarization (Figure 8A). Additionally, the fluorescence intensity of CD206, a marker of the M2 phenotype, significantly increased under CLMD intervention (Figure 8B). The RT‒qPCR results revealed that the relative mRNA expression of M1-associated proinflammatory factors (IL6, IL1B and TNF) increased significantly under LPS induction and decreased significantly under CLMD intervention (Figure 8C) (Lu et al., 2025). The relative mRNA expression of M2-associated anti-inflammatory factors (IL4, IL10, and TGFβ) was significantly increased under CLMD intervention (Figure 8D) (Zheng et al., 2025). Together, these results suggest that CLMD mitigates inflammatory responses by modulating the balance of M1/M2 polarization.

## 4 Discussion

This study used an integrated approach combining network pharmacology, machine learning, molecular dynamics, and *in vitro* experiments to clarify the primary mechanisms through which Chaihu-Longgu-Muli decoction (CLMD) is used to treat perimenopausal syndrome (PMS). Our findings revealed that the main active ingredients of CLMD (quercetin, kaempferol, and wogonin) target key inflammatory nodes, such as IL6, IL1B, and TNF, and regulate estrogen-related processes, the TNF signaling pathway, and cellular senescence. These modifications improved the inflammatory microenvironment and immune balance, providing a mechanistic explanation for the therapeutic effects of CLMD on PMS-related symptoms such as hot flashes, sleep disturbances, and mood changes.

The etiology of postmenopausal estrogen fluctuations is associated with inflammation–neuroimmune imbalance (Priyanka and Nair, 2020; Yu et al., 2025). Estrogen decline or fluctuation can activate peripheral immunity, increasing the levels of IL6, IL1B and TNF, thereby forming an inflammatory feedback loop (Guo et al., 2018; Yu et al., 2025). Clinical evidence suggests that estradiol levels are negatively correlated with the levels of inflammatory factors, such as TNF (Mruczyk et al., 2025). Moreover, the upregulation of inflammatory factors is closely linked to PMS-related clinical symptoms, such as depression, sleep disturbance, and cognitive impairment (Guo et al., 2018; Priyanka and Nair, 2020; Yu et al., 2025). On the basis of the intersection network of CLMD-active ingredient-disease targets, we identified 46 effective targets. The potential roles of CLMD are associated with Gene Ontology (GO) terms such as estrogen response element binding, aromatase activity, and estrogen metabolic process, as well as Kyoto Encyclopedia of Genes and Genomes (KEGG) pathways such as cellular senescence and the TNF signaling pathway. These findings suggest that CLMD might improve key PMS pathologies by modulating estrogen-related signaling and inflammatory pathways simultaneously (Chen et al., 2023; Crandall et al., 2023; Inayama et al., 2023; Santoro, 2016). Notably, on the basis of PPI network–assisted machine learning predictions, we further screened three key targets: IL6, IL1B and TNF. As inflammatory mediators, these targets are pivotal for systemic inflammation and central sensitization and are important pathological factors for vascular symptoms, mood disorders, and sleep problems (Deng et al., 2025; Jia et al., 2023; Muka et al., 2016; Tang et al., 2023; Zhang et al., 2024). The binding energies between the key active ingredients of CLMD (quercetin, kaempferol, and wogonin) and the aforementioned targets were all less than −5.0 kcal/mol. MD simulations revealed that the RMSD, Rg, SASA, and hydrion bond counts stabilized after approximately 50 ns and remained stable for at least 200 ns. Furthermore, the total binding free energies (MM/GBSA) fell within a favorable range, which supported the stable binding of quercetin to IL6, IL1B, TNF, kaempferol to TNF, and wogonin to TNF (Elekofehinti et al., 2021; Gong et al., 2025; Pronk et al., 2013).

At the cellular level, the lipopolysaccharide (LPS)-induced Raw 264.7 model reproduced inflammatory stress-driven abnormal proliferation and reactive oxygen species (ROS) accumulation. This was accompanied by a bias toward M1 polarization and excessive secretion of proinflammatory factors (Deng et al., 2025; Li et al., 2023; Sha et al., 2023; Yang et al., 2025). CLMD intervention significantly reduced the expression of proteins such as IL6, IL1B and TNF, as well as the level of ROS. IF and RT‒qPCR revealed decreased CCR7 and increased CD206 expression, suggesting a tendency toward a shift from the M1 phenotype to the M2 phenotype. These results demonstrated that CLMD can improve the inflammatory microenvironment by modulating M1/M2 polarization (Liao et al., 2025; Lu et al., 2025; Zhao et al., 2018). Taken together, these results validated the ability of CLMD to regulate the inflammatory environment through anti-inflammatory actions, antioxidant stress, and immune reprogramming. The enrichment of cellular senescence in our analyses further suggested that CLMD might buffer the interactive feedback between inflammation-driven aging phenotypes and endocrine fluctuations (Coperchini et al., 2025; Huang et al., 2019; Mishra and Brinton, 2018). Thus, this study highlights the key role of inflammatory cytokine networks in PMS and establishes the key pharmacodynamic effects of CLMD at key immune-inflammation regulatory nodes centered on IL6, IL1B and TNF. These findings provide a mechanistic rationale for alleviating hot flashes, sleep disturbances, and mood-related symptoms.

Female reproductive aging is a natural process, marked by a gradual decline in the number of follicles and the quality of oocytes (Lliberos et al., 2021). This process leads to the progressive loss of a woman’s reproductive capacity and ovarian function, ultimately resulting in perimenopausal syndrome. Studies have shown that as aging, the levels of pro-inflammatory cytokines (such as IL1β, TNFα, and IL6) in the serum and within the ovaries significantly increase (Lliberos et al., 2021). This feature is in line with one of the typical characteristics of cellular senescence, the increased secretion of senescence associated secretory phenotype (SASP) (Kumari and Jat, 2021). It is indicated that the inflammatory burden caused by the senescence of ovarian cells might play a crucial role in the occurrence and development of PMS.

The anti-inflammatory effects of CLMD mainly result from the synergistic action of multiple mechanisms, including inhibiting inflammatory factors, antioxidant effects, immune regulation. These effects may have a certain alleviating effect on the inflammatory burden related to perimenopause, such as chronic low-grade inflammation (Cybulska et al., 2025). However, the efficacy and persistence are often limited by the complexity of components, dosage, and individual differences. HRT is the main treatment method for PMS. Its main function is not to directly combat inflammation, but to regulate neuroendocrine, metabolic and circulatory systems by supplementing estrogen for alleviating systemic symptoms (Chen et al., 2023; Rozenberg et al., 2013). In terms of safety, studies have shown that long-term use of HRT is associated with an increased risk of breast cancer, endometrial cancer, venous thrombosis, and cardiovascular events (Rozenberg et al., 2013; Troia et al., 2025). The combination with CLMD is expected to reduce the reliance on HRT, thereby minimizing the risk of side effects (Wang and Yu, 2021). Furthermore, it is worth noting that the anti-inflammatory potential of CLMD may be also partially attributed to its direct regulation of the function of lipid rafts (Capozzi et al., 2023; Mattioli et al., 2023). This is because the active ingredients in CLMD contain flavonoids, such as quercetin and kaempferol. And flavonoids have been proven to effectively prevent the accumulation of lipid rafts, thereby reducing the production of pro-inflammatory cytokine (Kumazawa et al., 2006).

The advantages of this study are as follows: (1) Multiple machine learning algorithms (LASSO, SVM-RFE, and RF) were applied and cross-validated to identify key targets, increasing the predictive accuracy of network pharmacology (Luo et al., 2025; Wenjie et al., 2025; Xiao et al., 2025). (2) The combination of molecular dynamics simulations and MM/GBSA free energy calculations provided evidence of binding stability between key active ingredients and key targets (Luo et al., 2025; Vinothkanna et al., 2025). (3) The study used an *in vitro* inflammatory model induced by LPS to validate the AI-assisted prediction–functional verification strategy. However, several limitations have been noted: (1) PMS pathology involves a complex endocrine–neuroimmune network, but the current model focuses primarily on the inflammatory microenvironment and macrophage phenotype polarization. (2) Network pharmacology and machine learning algorithms rely on parameter settings. Although cross-validation has improved robustness, there is still potential for false negatives and false positives in target identification. (3) While *in vitro* research has validated the therapeutic mechanism of CLMD for PMS-related symptoms, additional confirmation requires further clinical studies.

In summary, the incidence of PMS increases with age and markedly impairs quality of life. Owing to the risks and limited use of hormone replacement therapy (HRT), multitarget strategies with fewer adverse effects, including replacement or combination approaches, are highly valuable (Chen et al., 2023; Crandall et al., 2023; Inayama et al., 2023; Santoro, 2016; Wislowska-Stanek et al., 2025). The regulatory mechanism revealed in this study, in which IL6, TNF, and IL1B serve as key targets. Moreover, anti-inflammatory, antioxidant, and immune reprogramming processes constitute key mechanisms. This study provides mechanistic support for the overall efficacy of CLMD in alleviating hot flashes, sleep disturbances, and mood fluctuations. This mechanism also provides the basis for individualized therapeutic strategies that could be used in combination with or as an alternative to HRT.

## 5 Conclusion

This study used an integrated approach combining network pharmacology, machine learning, molecular dynamics (MD), and *in vitro* experiments to fully reveal the mechanisms by which Chaihu-Longgu-Muli decoction (CLMD) treats perimenopausal syndrome (PMS). Combining data from the TCMSP, BATMAN-TCM, and other databases allowed us to construct an ingredient–target–disease network and identify key inflammatory hubs centered on IL6, IL1B, and TNF. The enrichment analyses revealed critical pathways (including those involved in cellular senescence), indicating that CLMD modulates the inflammatory microenvironment via these intervention routes. MD simulations revealed that the active ingredients formed conformationally stable complexes with the identified targets, supporting the structural and energetic rationality of network pharmacology predictions. *In vitro* functional assays confirmed that CLMD significantly downregulates IL6, IL1B, and TNF, reduces ROS accumulation, and promotes macrophage polarization from the M1 phenotype to the M2 phenotype. These results were consistent with the predicted anti-inflammatory actions. Convergent evidence from functional assays and pathway enrichment corroborates the proposed mechanism. Taken together, these findings suggest that CLMD exerts synergistic effects on multiple ingredients, modulating the cytokine network and inflammatory environment. These findings provide mechanistic support for its ability to alleviate PMS-associated vascular, sleep, and mood-related symptoms.

## Data Availability

The raw data supporting the conclusions of this article will be made available by the authors, without undue reservation.
